# Digital Decoding of Single Extracellular Vesicle Phenotype Differentiates Early Malignant and Benign Lung Lesions

**DOI:** 10.1002/advs.202204207

**Published:** 2022-11-17

**Authors:** Junrong Li, Abu A. I. Sina, Fiach Antaw, David Fielding, Andreas Möller, Richard Lobb, Alain Wuethrich, Matt Trau

**Affiliations:** ^1^ Centre for Personalised Nanomedicine Australian Institute for Bioengineering and Nanotechnology (AIBN) The University of Queensland Brisbane QLD 4072 Australia; ^2^ Department of Thoracic Medicine Royal Brisbane and Women's Hospital Herston QLD 4029 Australia; ^3^ Tumour Microenvironment Laboratory QIMR Berghofer Medical Research Institute Herston Queensland 4006 Australia; ^4^ School of Chemistry and Molecular Biosciences The University of Queensland Brisbane QLD 4072 Australia

**Keywords:** lung cancer screening, plasmonic nanostructures, small extracellular vesicles, surface‐enhanced Raman scattering

## Abstract

Accurate identification of malignant lung lesions is a prerequisite for rational clinical management to reduce morbidity and mortality of lung cancer. However, classification of lung nodules into malignant and benign cases is difficult as they show similar features in computer tomography and sometimes positron emission tomography imaging, making invasive tissue biopsies necessary. To address the challenges in evaluating indeterminate nodules, the authors investigate the molecular profiles of small extracellular vesicles (sEVs) in differentiating malignant and benign lung nodules via a liquid biopsy‐based approach. Aiming to characterize phenotypes between malignant and benign groups, they develop a single‐molecule‐resolution‐digital‐sEV‐counting‐detection (DECODE) chip that interrogates three lung‐cancer‐associated sEV biomarkers and a generic sEV biomarker to create sEV molecular profiles. DECODE capturessEVs on a nanostructured pillar chip, confines individual sEVs, and profiles sEV biomarker expression through surface‐enhanced Raman scattering barcodes. The author utilize DECODE to generate a digitally acquired sEV molecular profiles in a cohort of 33 people, including patients with malignant and benign lung nodules, and healthy individuals. Significantly, DECODE reveals sEV‐specific molecular profiles that allow the separation of malignant from benign (area under the curve, AUC = 0.85), which is promising for non‐invasive characterisation of lung nodules found in lung cancer screening and warrants further clinincal validaiton with larger cohorts.

## Graphical abstract

Digital small extracellular vesicle (sEV) counting detection (DECODE) chip—a nanostructured array integrated with surface‐enhanced Raman scattering to capture and characterize single sEVs by forming a binary unit (DECODE pixel)—detects phenotypic differences in plasma sEVs of patients with benign or malignant lung lesions. The digital analyses of liquid biopsy by DECODE are promising for noninvasive early‐stage lung cancer screening.

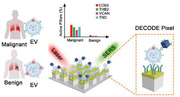

## 1 Introduction

Lung cancer is associated with high mortality among both men and women, constituting almost 25% of all cancer‐related deaths.^[^ ^1^ ^]^ Accurate lung cancer screening is regarded as the most effective way to identify earlier stages of the disease and reduce the high mortality of lung cancer (5 year survival of 18%).^[^ ^2^, ^3^ ^]^ Although focused lung cancer screening programs are being trailed, the routine integration of effective screening into the clinical setting still has some issues to overcome. This is because the current diagnostics have 1) inadequate capability of evaluating indeterminate lung nodules;^[^ ^4^ ^]^ 2) limited applicability for frequent sampling;^[^ ^5^ ^]^ and 3) expensive testing of large groups.^[^ ^6^ ^]^ Specifically, low‐dose computer tomography (LDCT) as the gold standard for lung cancer assessment faces a dilemma in identifying both malignant and benign nodules, because of their similar features (e.g., size and attenuation). For instance, in a focused screening trial with LDCT on 2106 high‐risk individuals, LDCT found a suspected lung lesion of 59.7%; however, only 2.5% of these positive individuals had lung cancer, as revealed by follow‐up testing.^[^ ^7^ ^]^ To confirm the cancer diagnosis, bronchoscopies are conducted to immunohistochemically analyze the extracted tissue samples. However, the use of tissue biopsy to assist in lung cancer screening is not amenable for frequent sampling required for patient monitoring, and has the risk of potentially “seeding” the cancer cells to other sites.^[^ ^8^ ^]^ Furthermore, limited by the high cost and the requirement of professional staff, LDCT is mostly performed for high‐risk individuals (e.g., smokers or past smokers), which is likely to exclude an increasing number of nonsmoking‐related lung cancers.^[^ ^9^, ^10^ ^]^ Therefore, an accurate, minimally invasive lung cancer screening approach that is applicable to large‐scale monitoring and capable of identifying early malignant and benign lesions is yet an unmet clinical need.

To allow accurate lung cancer screening without the limitations of tissue biopsy, molecular profiling of circulating biomarkers in patients’ bodily fluids (e.g., blood or urine) can be a promising strategy for classifying malignant and benign nodules. Playing a key role in cancer cell communication, the circulating small extracellular vesicles (sEVs) and their phenotypic fingerprinting are gaining momentum for cancer screening and treatment monitoring.^[^ ^11^, ^12^ ^]^ For instance, the analysis of protein biomarkers on sEVs using a thermophoretic aptasensor has been reported to accurately monitor and predict treatment response in metastatic breast cancer, which highlighted the clinical utility of sEVs in cancer management.^[^ ^13^ ^]^ Small EVs display tetraspanin proteins (e.g., CD9, CD63, and CD81) on the surface that differentiate them from other extracellular particles, such as apoptotic bodies and protein macroaggregates. In the context of lung cancer screening, sEVs enriched with adhesion markers including thrombospondin 2 (THBS2), versican (VCAN), and tenascin C (TNC) were found to closely indicate lung cancer incidence and shown to accurately distinguish tumor tissue and normal tissue.^[^ ^14^ ^]^ THBS2 is a matricellular glycoprotein that affects proliferation and extracellular matrix (ECM) modeling, and it has been linked to severe thrombosis in lung cancer patients.^[^ ^15^ ^]^ VCAN is involved in cellular recognition and interferes with cell adhesion to the extracellular matrix.^[^ ^16^ ^]^ TNC is connected to tissue morphogenesis and lung metastasis.^[^ ^17^ ^]^ We reasoned that creating a molecular profile based on the individual expression of THBS2, VCAN, TNC, and CD63 on sEVs could distinguish blood of patients with benign and malignant lung lesions. Notably, the diagnostic use of such sEV molecular profiles to differentiate malignant and benign lung nodules remains unexplored. Additionally, the practical utility of sEVs as biomarkers is complicated by the sEV heterogeneity and the trace abundance of cancer‐specific sEVs in circulation.^[^ ^18^, ^19^, ^20^ ^]^ As a result, a highly sensitive technology that allows multi‐biomarker detection for heterogeneous molecular profiling is needed to translate sEV analysis for lung cancer screening.

Conventional techniques for sEV analysis (e.g., enzyme‐linked immunosorbent assay (ELISA)) enable bulk analysis of sEVs. However, the limited multiplexing capability and the low sensitivity hinder their usage in detecting rare cancer‐specific sEVs, especially for accurately discriminating malignant and benign nodules. Advances in nanomaterial‐based systems and biosensors ushered in a suite of methods that are capable of multiplexed analysis of plasma sEVs without requiring prior sEV isolation.^[^ ^13^, ^21^ ^]^ The unique physicochemical properties of nanomaterials enable multiple biomarker detection in trace quantities. Particularly, by leveraging the strong electromagnetic coupling effect of nanostructures to remarkably enhance inelastic scattering and the extremely narrow Raman spectrum, nanomaterials and surface‐enhanced Raman scattering (SERS) integrated platforms can offer the sensitivity and highly multiplexing option ideal for sEVs’ profiling.^[^ ^22^ ^]^ Our work capitalizes on a nanopillar array and superplasmonic Au–Ag alloy nanoboxes that provide single‐nanoparticle SERS activity for digital readout of sEV profiles. Nanopillar array and nanoboxes combined into a digital SERS platform are capable of individually capturing and directly profiling single sEVs, providing critical advantages for detecting sEV phenotypic differences in malignant and benign lung nodules. Our digital SERS platform is more sensitive than previously reported SERS platforms for sEV analysis (**Table** **1**) and facilitates in situ multiplexing that is not possible with other sensitive sEV readouts such as fluorescence, surface plasmon resonance, and electrochemical nanotechnologies. Future technological advances in confocal Raman microscopy for faster chip readouts could further support the translation of digital sEV counting detection (DECODE) into the clinical setting.

**Table 1:** Comparison of reported techniques for multiplex profiling of sEVs

Detection	Principle	Multiplexing/sEV targets	Limit of detection	Ref.
Digital SERS (DECODE)	Confinement of single sEV on nanopillar, labeling with SERS nanotags	In situ TNC, CD63, THSB2, and VCAN	12 sEVs µL^−1^	This study
SERS	Magnetic bead‐based isolation of sEVs, labeling with SERS nanotags	In situ CD63, GPC‐1, EpCAM, and CD44V6	2300 sEVs µL^−1^	[29]
Digital fluorescence	Encapsulation of single sEV‐magnetic bead complexes in droplets, enzymatic amplification of signal	Parallel reactions CD63, GPC‐1	10 sEVs µL^−1^	[30]
Electrochemical	Magnetic bead‐based isolation of sEVs, enzymatic amplification of electrochemical signal	Parallel reactions CD63, CD9, CD81, EGFR, EpCAM, CD24, and GPA33	10 sEVs µL^−1^	[31]
Surface plasmon resonance	Plasmonic shift upon binding of nanorods and nanosphere to sEVs	In situ CD81, CD9, and EphA2	0.23 ng µL^−1^	[32]

Due to the clinical need of differentiating malignant and benign lung lesions, we were motivated to develop a noninvasive, blood‐based method for decoding the molecular profile of cancer‐specific sEVs with single EV precision. This blood‐based sEV profiling approach avoids the limitations of traditional tissue biopsy. We have previously shown that SERS can detect single cytokines in plasma samples and demonstrated an integrated SERS microfluidic system for bulk sEV phenotyping in treatment monitoring of late‐stage melanoma patients.^[^ ^23^, ^24^ ^]^ In the current study, we design a digital sEV counting and detection (DECODE) chip to acquire sEV molecular profiles for accurate lung cancer diagnosis. DECODE capitalizes on a gold‐toped silicon nanopillar array to confine individual sEVs using Poisson distribution together with superplasmonic Au–Ag alloy nanoboxes for signal readout.^[^ ^25^ ^]^ Counting of sEV‐activated nanopillars creates a digital readout of single sEV molecular profiles that avoid quantification bias due to SERS signal intensity fluctuation. Importantly, we investigated the possibility of using sEV molecular profiles (i.e., CD63, THBS2, VCAN, and TNC) produced by DECODE to indicate malignant and benign lung lesions. Encouragingly, DECODE correctly classified 33 individuals with early malignant lung lesion (i.e., stages I and II), benign lung lesions, and healthy participants based on their specific sEV molecular profiles in plasma. With the capability of multiparameter profiling trace level of lung‐cancer‐specific EVs, we suggest that DECODE has the potential as a screening tool to accurately identify suspected lung lesions without invasive tissue biopsy and can benefit a wider population.

## 2 Results

### 2.1 DECODE Acquiring sEV Molecular Profiles

**Figure 1:**
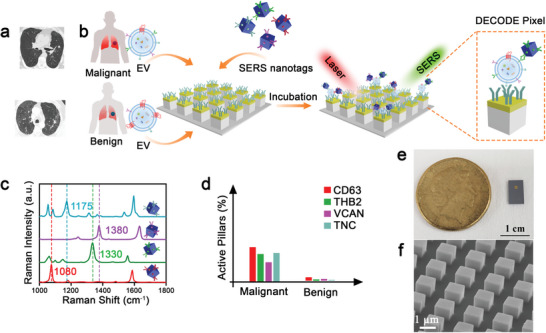
Digital sEV counting detection (DECODE) chip to differentiate malignant and benign lung nodules. a) CT images of patients with malignant (top) and benign (bottom) lung lesions. b) Schematic of DECODE to count and phenotype sEVs using anti‐TNC antibody conjugated nanopillar array and nanobox‐based SERS barcodes that target sEV expression of CD63, THSB2, VCAN, and TNC. c) SERS spectra of Raman reporters with characteristic Raman signals: 2,7‐mercapto‐4‐methylcoumarin (MMC, 1175 cm^−1^), 2,3,5,6‐tetrafluoro‐4‐mercaptobenzoic acid (TFMBA, 1380 cm^−1^), 5,5‐dithiobis‐2‐nitrobenzoic acid (DTNB, 1330 cm^−1^), and 4‐mercaptobenzoic acid (MBA, 1080 cm^−1^). d) Representative DECODE‐enabled sEV molecular profiles of malignant and benign patients. e) Photograph of the DECODE chip. f) Tilted scanning electron microscope (SEM) image of the nanopillar array.

To screen a suspected lung disease, chest imaging is typically ordered in the clinics. However, the accurate classification of a lung lesion into benign and malignant is difficult as the positron emission tomography (PET) and computer tomography (CT) images may show similar features (**Figure** **1**a). To correctly diagnose indeterminate lung nodules, subsequent immunohistochemical staining and morphological analysis of a tissue sample are required. As an alternative to this invasive approach in diagnosing lung cancer, we hypothesized that circulating sEVs derived from patient plasma carry specific molecular profiles to classify malignant and benign lung nodules. Specifically, we selected a panel of protein biomarkers expressed on sEV surfaces to constitute a lung sEV molecular profile, including CD63, THSB2, VCAN, and TNC. CD63 was used to as a generic sEV marker. As the increased levels of THSB2, VCAN, and TNC on sEVs were found to indicate lung adenocarcinoma patients, we combined these biomarkers and explored their usage as a signature in identifying malignant and benign lung lesions.^[^ ^14^ ^]^

**Figure 2:**
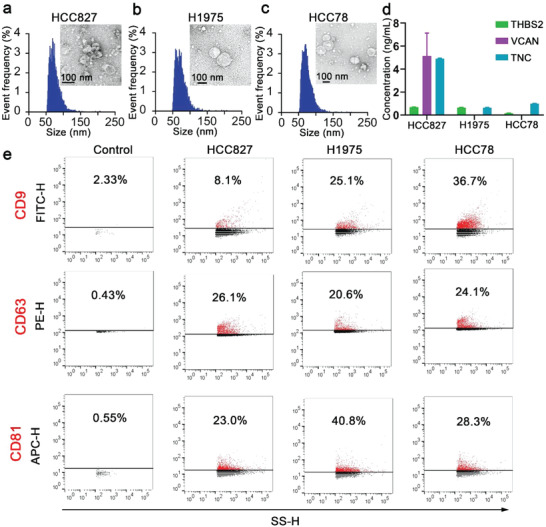
Characterization of sEVs derived from lung cancer cell lines. Size distribution analysis and TEM image of a) HCC827, b) H1975, and c) HCC78 cell line sEVs. d) sEV surface biomarker expressions of THBS2, VCAN, and TNC by conventional ELISA. e) Nanoflow cytometry of sEVs’ expression of tetraspanins (CD9, CD63, and CD81).

DECODE enables the phenotypic profiling of sEVs by integrating a nanopillar array and gold–silver alloy nanobox‐based SERS barcodes that possessed single particle activity (Figure 1b). Plasma sEVs were purified through size exclusion chromatography (SEC) and captured on DECODE's nanopillar array functionalized with anti‐TNC antibody to bind lung‐cancer‐associated sEVs. Among the targeted lung‐cancer‐associated sEV makers (i.e., THSB2, VCAN, and TNC), we selected TNC for sEV capture because it was consistently expressed across the tested lung cancer cell lines (**Figure** **2**d) used in this study. The lung‐cancer‐associated sEVs were enriched and captured on the nanopillar array with anti‐TNC antibody, followed by reading the expression of THBS2, VCAN, TNC, and general sEV marker CD63 through the nanobox‐based SERS barcodes. We expected that the antibody‐based affinity enrichment and reading of sEV profiles enabled the discrimination of sEVs’ phenotypes for individuals with benign and malignant lung lesions.

To allow a digital multiplex profiling of sEV surface biomarkers, the SERS barcodes utilize ≈80 nm nanoboxes (Figure S1, Supporting Information) to co‐conjugate with different pairs of Raman reporters and antibodies for target identification. Particularly, Raman reporters that possess characteristic Raman signals (i.e., 4‐mercaptobenzoic acid (MBA) at 1080 cm^−1^, 5,5‐dithiobis (2‐nitrobenzoic acid) (DTNB) at 1330 cm^−1^, 2,3,5,6‐tetrafluoro‐4‐mercaptobenzoic acid (TFMBA) at 1380 cm^−1^, and 2,7‐mercapto‐4‐methylcoumarin (MMC) at 1175 cm^−1^) are employed to indicate the sEV surface expression of CD63, THBS2, VCAN, and TNC, respectively (Figure 1c). The nanoboxes are able to load multiple antibodies on their surfaces, and the similar size of nanoboxes and sEVs is likely to preferentially yield in highly efficient single binding events. The construction of nanopillar‐sEV‐SERS barcodes forms a DECODE pixel, a binary unit that is either activated when sEVs are present or deactivated in the absence of sEVs. By counting the activated DECODE pixels for each marker and representing the four markers together, an sEV molecular profile specific for each patient is created to assist the accurate diagnosis of malignant or benign lung lesions without the need for expensive and time‐consuming imaging modalities, or invasive tissue biopsies (Figure 1d).

To perform DECODE analysis for lung lesion, controlling the sEV concentration to follow a Poisson distribution is critical to the measurement. At a ratio of one sEV per ten nanopillars, the probability of one or zero sEV on an individual nanopillar is higher than 99%, which supports the digital counting principle.^[^ ^26^ ^]^ In this regard, we applied ≈2.5 × 10^4^ EVs on the nanopillar array with 2.5 × 10^5^ pillars to activate maximum 10% nanopillars for accurate quantification. Furthermore, rationally regulating nanopillar spacing is necessary for effective confocal SERS mapping. Given the approximate resolution of ≈1 µm on SERS image, we fabricated the nanopillar array to have a separation of 1 µm to enable the acquisition of Raman spectra on individual nanopillars without overlap from adjacent nanopillars (Figure 1e,f). Additionally, to reduce potential nonspecific SERS signals on the silicon substrate, the nanopillar height is designed to be 1 µm high and thereby allowing the selective collection of signals from the top of the nanopillars. Taken together, by applying sEVs with designated concentrations on precisely fabricated nanopillars, DECODE strategy facilitated SERS phenotyping of single sEVs for the generation of molecular profiles. Particularly, the digital “yes/no” SERS counting principle of DECODE has the key advantage of accurate sEV measurement via providing multi‐biomarkers and circumventing reproducibility issues of SERS signals encountered with ensemble measurements.

### 2.2 sEV Characterization

We first characterized the physical and biological properties of lung cancer cell line (i.e., HCC827, H1975, and HCC78) derived sEVs, including morphology, size distribution, and expression of lung‐cancer‐associated biomarkers (i.e., THBS2, VCAN, and TNC) and canonical tetraspanin (i.e., CD9, CD63, and CD81) to establish and study the validity of cell line sEVs for lung cancer screening (Figure 2). Transmission electron microscope (TEM) images (insets of Figure 2a–c) showed the presence of sEVs with a lipid bilayer structure and size range between 30 and 150 nm. Nanoflow cytometry measurement (Figure 2a–c) further demonstrated a size distribution of sEVs from ≈50 to 200 nm, fitting in the typical size range of sEVs. These data also suggested that the cell line lung cancer sEVs shared similar physical features. However, different to the morphological similarities was the sEV surface expression of lung‐cancer‐associated biomarkers (i.e., THBS2, VCAN, and TNC) (Figure 2d). We found diverse expression levels of proteins on sEVs between cell lines (Figure 2d). Specifically, HCC827‐derived sEVs expressed relatively low levels of THBS2 but high levels of both VCAN and TNC, whereas H1975 and HCC78 cell line sEVs displayed low THBS2 expression, absence of VCAN, and low TNC expression. Furthermore, nanoflow cytometry profiling through fluorophore‐labeled immunostaining validated sEV identity and indicated the heterogeneous expression of tetraspanin biomarkers (i.e., CD9, CD63, and CD81) on sEV surfaces, in which CD63 and CD81 had relatively higher levels for these three cell lines (Figure 2e).

### 2.3 DECODE Phenotyping sEVs Derived from Lung Cancer Cell Lines

**Figure 3:**
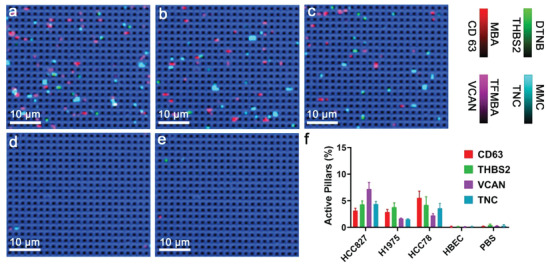
DECODE‐specific profiling lung cancer cell‐line‐derived sEVs. SERS mapping images of the sEVs collected from a) HCC827, b) H1975, c) HCC78, d) HBEC cell lines, and e) PBS control. f) Corresponding sEV molecular profiles from panels (a)–(e).

We hypothesized that the ability to generate specific sEV molecular profiles showing CD63, THBS2, VCAN, and TNC expressions is essential for distinguishing malignant and benign lung nodules. We thus first evaluated the DECODE chip in phenotyping sEVs derived from three lung cancer cell lines (i.e., HCC827, H1975, and HCC78), an untransformed normal human bronchial epithelial cell (HBEC), and phosphate‐buffered saline (PBS) control. We reasoned that the biomarker profile of the lung cancer cell‐derived sEVs resembles that of sEVs derived from a malignant lung lesion, while the HBEC sEVs’ profile corresponds to the profile of a benign lung lesion. DECODE was designed to display the confocal SERS images by revealing bright spots in four colors (i.e., red, green, purple, and cyan) on a blue grid, in which the four colors corresponded to four biomarker expressions (i.e., CD63, THBS2, VCAN, and TNC) and the blue grid indicated the silicon signals (520 cm^−1^). As anti‐TNC antibody‐functionalized nanopillars enrich cancer‐associated sEVs, we expected DECODE to provide an sEV molecular profile with higher expressions of CD63, THBS2, VCAN, and TNC in the three lung cancer cell lines and low/negligible biomarker expression from HBECs and PBS controls. Consistent with our assumptions, the SERS images of lung cancer cell‐line‐derived sEVs showed abundant bright spots (**Figure** **3**a–c), whereas HBEC‐derived sEVs and PBS showed only a few bright spots (Figure 3d,e). These results suggested the high specificity of DECODE in detecting lung‐cancer‐associated sEVs.

We were then motivated to explore the capability of DECODE to create and define sEV molecular profiles. By counting CD63, THSB2, VCAN, and TNC signals per nanopillar array, we calculated the active pillar percentage of each biomarker that reflected the sEV molecular profiles. As expected, following successful anti‐TNC antibody capture of sEVs on DECODE, HCC827‐, H1975‐, and HCC78‐derived sEVs displayed characteristic molecular profiles with varying levels of the four biomarkers (Figure 3f). Although a direct comparison of DECODE with the results from bulk sEV analysis (Figure 2) is difficult, DECODE provides single‐molecular level sensitivity to reveal traces of sEVs’ phenotypic differences that are unlikely to be detected by bulk analysis. Specifically, nanoflow cytometry required labeling sEVs with multiple fluorophore‐conjugated antibodies per sEVs and analyzing ≈10^8^ sEVs mL^−1^ per measurement. ELISA provided an average signal readout of the sEV sample, which was likely to conceal subtle sEVs’ phenotypic differences in the signal noise. A similar finding of insufficient ELISA sensitivity to measure sEV surface protein biomarkers has been reported.^[^ ^27^ ^]^ Furthermore, in comparison to nanoflow cytometry and ELISA, DECODE also demonstrated the intrinsic multiplexing advantage of reading all biomarkers in trace amount of sEV samples. As for the HBEC‐derived sEVs and PBS, DECODE showed no identifiable molecular profiles due to the failure of capturing TNC‐expressed sEVs.

Taken together, DECODE chip showed the ability to specifically profile lung cancer sEVs and provided unique sEV molecular profiles to indicate different cell lines. We therefore hypothesized that the use of DECODE could produce sEV molecular profiles for lung cancer patient screening, and more importantly, classification of malignant and benign lung lesions.

### 2.4 DECODE Detection Sensitivity for sEVs Derived from Lung Cancer Cell Line

**Figure 4:**
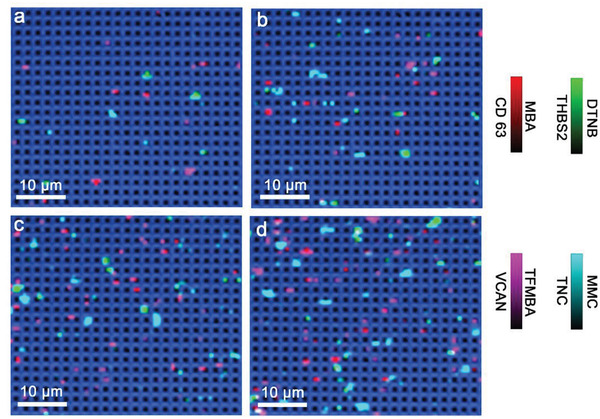
DECODE chip for sensitive profiling of sEVs derived from HCC827 cells. SERS images of sEVs at a number of a) 1.25 × 10^2^, b) 1.25 × 10^3^, c) 1.25 × 10^4^, and d) 2.5 × 10^4^ particles.

Due to the low levels of cancer‐specific sEVs in clinical samples, a sensitive platform that can clearly reveal sEV molecular profiles is of great importance to accurate lung cancer screening. To assess the sensitivity of DECODE in profiling sEVs, we titrated sEVs derived from HCC827 cells with designated particle concentrations to comply Poisson distribution and monitored the activated pillars on SERS images. Specifically, we applied sEVs at a number of 1.25 × 10^2^, 1.25 × 10^3^, 1.25 × 10^4^, and 2.5 × 10^4^ particles on DECODE chip, which corresponded to a maximum 0.05%, 0.5%, 5%, and 10% of activated pillars. As shown in **Figure** **4**, with a higher sEV number captured on DECODE chip, an increasing proportion of pillars is activated for all four biomarkers. To further allow quantitative profiling of each biomarker and evaluating the dynamic range, we counted the activated pillars and plotted the logarithmic transformation of the activated pillar percentage versus sEV concentration. As suggested by the linear relationship curves in Figure S2 (Supporting Information), DECODE facilitated the sensitive sEV profiling via CD63, THBS2, VCAN, or TNC with the use of sEVs down to a number as low as 1.25 × 10^2^ particles, which correspond to an sEV concentration of ≈12 particles µL^−1^. At this limit of detection level (i.e., ≈12 sEVs µL^−1^), it was not possible to validate the accuracy or precision of the DECODE chip, because of the insufficient sensitivity for the commercially available techniques (e.g., ELISA and nanoflow cytometry) and the difficulty to obtain a standard sEV sample with a known concentration of TNC expression. However, the concept of digitally counting sEVs on nanopillars is advantageous as it provides a strategy for accurate and absolute quantification, theoretically not requiring the establishment of a calibration curve or use of reference materials.^[^ ^28^ ^]^

As compared to the developed technologies for sEV profiling, DECODE demonstrated the advantages of allowing both highly sensitive and in situ multiplexed detection of trace populations of tumor‐derived sEVs, which is critical to pinpoint the subtle phenotypic changes between malignant and benign lung nodules. Table 1 summarizes several reported techniques to detect sEVs. The traditional SERS intensity‐based assay delivered a low sensitivity (i.e., 2300 sEVs µL^−1^) that restricted its use in detecting the trace level of tumor‐derived sEVs.^[^ ^29^ ^]^ For the digital fluorescence and electrochemical techniques, they improved the detection sensitivity by introducing enzymatic amplification, but the multiplex analysis had to rely on the parallel detection of sEV replicate samples.^[^ ^30^, ^31^ ^]^ Similarly, the surface plasma resonance strategy allowed a limited multiple biomarker detection due to the overlap of wavelength in scattered light.^[^ ^32^ ^]^

### 2.5 DECODE Profiling sEVs Derived from Clinical Samples

Having demonstrated the specificity and sensitivity of DECODE chip in sEV profiling, we now utilized DECODE to compare sEV molecular profiles of 33 clinical samples derived from the plasma of patients with malignant lung nodules (M1–M11), benign lung nodules (B1–B11), and healthy subjects (H1–H11). We expected that sEVs secreted from benign and malignant lung tissues into circulation can be distinguished by the differences in sEV expression of the four targeted markers in plasma. To test our hypothesis that specific sEV molecular profiles may help classify patients and diagnose lung cancer, we incubated 2.5 × 10^4^ sEVs on DECODE chip to fulfill Poisson distribution, followed by conducting SERS mapping and counted each surface‐biomarker‐activated pillar to acquire sEV molecular profiles.

**Figure 5:**
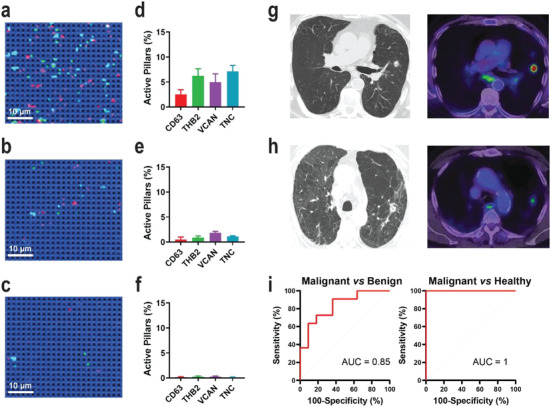
DECODE phenotyping clinical plasma‐derived sEVs. Representative SERS images of sEVs derived from a) malignant lung cancer (M1), b) benign lung cancer (B1), and c) healthy control (H1). Molecular profiles of sEVs derived from d) M1, e) B1, and f) H1. Corresponding CT (left) and PET (right) images of g) M1 and h) B1. ROC curves of i) malignant versus benign (left) and malignant versus healthy (right).

For malignant lung nodules, sEV molecular profiles typically showed a relatively high level of the four biomarkers. On the other hand, benign lung nodules and healthy controls showed sEV molecular profiles with lower and negligible biomarker expressions, respectively. As a demonstration, **Figure** **5** shows representative sEV molecular profiles of one malignant lung nodule (M1), one benign lung nodule (B1), and one healthy (H1) plasma samples. The sEV molecular profiles of M2–M11, B2–B11, and H2–H11 are shown in Figures S3–S5 (Supporting Information), respectively. Specifically, the M1‐derived sEV SERS image demonstrated a high proportion of illuminated pillars in four colors (Figure 5a), thus suggesting a high expression of CD63, THBS2, VACAN, and TNC. On the contrary, B1‐ and H1‐derived sEV SERS images showed much less activated pillars (Figure 5b,c). Consistent with the visual SERS images, the sEV molecular profile quantitatively displayed a higher active pillar percentage of M1 than B1 and H1 (Figure 5d–f).

Importantly, the sEV molecular profile revealed by DECODE chip provided a differentiation between benign and malignant lung nodules. For comparison, the corresponding PET/CT images of M1 and B1 showed similar lung nodule features, not allowing for easy distinction of malignant and benign characteristics (Figure 5g,h). While the PET scan showed more metabolic activity in M1 than in the benign case, in neither case was the reporting nuclear physician able to determine unambiguously that one nor the other were definitively malignant or benign. For M1, it was reported that there were some benign features such as slightly mottled appearance of the nodule, which could indicate a benign granulomatous process. For B1, the lung nodule was reported as mild to moderately avid suggesting an inflammatory process; however, the nodule had been persistent over time and was enlarging, not ruling out a malignancy.

Intriguingly, DECODE demonstrated diverse biomarker expression levels of sEV molecular profiles among benign and malignant lung nodules, reflecting the intrapatient heterogeneity and highlighting the need of multi‐biomarker analysis to achieve accurate diagnosis. For instance, M2 sEV molecular profile had similar levels of CD63, THBS2, VCAN, and low level of TNC, whereas M3 sEV molecular profile significantly upregulated the levels of CD63, THBS2, and VCAN, but not TNC. On the other hand, B2 sEV molecular profile displayed low levels of all four biomarkers, but B4 sEV molecular profile demonstrated higher THBS2 and VCAN expressions and lower TNC level than CD63. Overall, malignant lung nodule‐associated sEVs derived from M2–M11 plasma (Figure S3, Supporting Information) typically possessed higher biomarker expression than those from B2–B11 (Figure S4, Supporting Information) and H2–H11 (Figure S5, Supporting Information), which indicated the possibility of sEV molecular profiles in identifying each class.

Ultimately, we statistically assessed the performance of DECODE‐produced sEV molecular profiles in diagnosing malignant lung nodule, benign lung nodule, or healthy controls. We first utilized the binary logistic regression on the sEV molecular profiles of malignant and benign lung nodules. The receiver operating characteristic (ROC) curve had an area under the curve (AUC) score of 0.85 (Figure 5i), which suggested the capability of sEV molecular profiles in separating malignant from benign nodules. We further tested the power of sEV molecular profiles in a direct lung cancer diagnosis by assessing lung cancer (i.e., malignant) and healthy participants. As suggested by the ROC curve with the AUC of 1.00, the generated sEV molecular profile had a high confidence in screening malignant lung nodules from healthy people. Collectively, DECODE showcased the ability to phenotype plasma‐derived EVs, and the unique EV molecular profiles had the potential to recognize malignant, benign lung nodules, or healthy subjects.

## 3 Discussion

Lung cancer mortality can be significantly reduced with the use of effective screening tools.^[^ ^33^ ^]^ CT and PET imaging of a suspicious lesion in combination with immunohistochemical analysis of a tissue sample extracted from the lesion are the current gold standard for lung cancer diagnosis. However, this approach has significant limitations such as late stage detection, costly and centralized infrastructure, and focusing on groups at high risk of lung cancer, thereby neglecting an increasing number of “atypical” cancers (e.g., nonsmokers).^[^ ^9^, ^10^ ^]^ Moreover, taking a tissue biopsy is an invasive procedure that requires a surgical intervention to extract tumor material from the lung and has the risk of spreading the tumor.^[^ ^8^ ^]^ Such an invasive approach is not amendable for monitoring or screening that would be required to evaluate if a person has a benign or malignant lung lesion. Our DECODE platform demonstrates a blood‐based screening approach that could significantly alleviate patient burden and reduce mortality by enabling early intervention.

Using CT or LDCT only without tissue sampling is generally not an applicable approach to discern benign and malignant lung lesions. Large studies have shown the power of CT and LDCT screening for lowering lung‐cancer‐related deaths. In the National Lung Screening Trial (NLST) in America with 53 454 high‐risk individuals, an annual CT screening resulted in a 20% of reduction in lung cancer mortality.^[^ ^34^ ^]^ Similarly, in the Nederlands–Leuvens Longkanker Screenings Onderzoek (NELSON) study, LDCT on 15 789 former or current smokers (aged 50–74) was performed four times over 5.5 years, and found to reduce cancer‐related mortality by 24% compared to no screening.^[^ ^33^ ^]^ However, both studies reported a high rate of false‐positive results (i.e., 26.3% in NLST and 19.8% in NELSON).^[^ ^35^ ^]^ LDCT scans frequently detect small long nodules (≈10 mm) that are confirmed to be benign upon more detailed analysis, which contributes significantly to the false‐positive results and thereby increases patient burden to hospitals and impacts on the quality of life for those individuals.^[^ ^4^, ^36^ ^]^ Using LDCT and the baseline nodule count also failed to differentiate lung nodules, it was therefore recommended to molecularly profile the nodules separately.^[^ ^37^ ^]^

To assist accurate lung cancer screening, an effective approach is directly acquiring the molecular profile of each patient instead of relying on the similar features of nodules (e.g., size and location) obtained in CT/LDCT. Furthermore, screening should be applicable and easily extendable to a larger population beyond solely focusing on smokers or former smokers. A noninvasive and cost‐efficient test for multisampling and routine monitoring is most promising in catching the malignancy early. This will help the clinician to deliver the patient with a tailored treatment plan that maximizes therapy success, while reducing side effects and healthcare costs.

The recognition of sEVs for cancer diagnosis has increased as they carry a similar phenotype to their cell of origin.^[^ ^11^, ^12^ ^]^ We hypothesized that the analysis of cancer‐associated sEVs to extract patient‐specific molecular profiles might help identifying malignant or benign lung nodules. With the advancement of technologies for sEV analysis, it has become evident that sEVs are heterogeneous, even when secreted from the same cells.^[^ ^19^, ^20^ ^]^ Moreover, cancer‐cell‐secreted sEVs are highly diluted in circulation due to the mixing with sEVs from other cells.^[^ ^11^ ^]^ To tackle the aforementioned challenges, highly sensitive analytical strategies to decode the subtle phenotypic changes of rare cancer‐associated sEVs for patient screening are required. With bulk measurements such as conventional ELISA, an average sEV signal is obtained, which can be problematic as the cancer‐associated signal can be overwhelmed in the bulk noise. In contrast, DECODE chip interrogates sEVs with single particle resolution to discern the phenotypic changes of benign and early malignant lung nodules. Key features of our work include the digital counting SERS design for single sEVs, sEV biomarker discovery, and unique clinical validation of DECODE chip on an utmost challenging patient sample cohorts consisting of early‐stage malignant and benign lung nodules. Compared to the traditional SERS intensity‐based assays, the digital counting SERS design avoids the Raman signal fluctuations for accurate sEV detection. The use of SERS nanotags to label and generate indirect signals provides specific protein biomarker detection on sEV surfaces. For intrinsic SERS signals (i.e., label‐free SERS) of sEVs, it is very difficult to discern the differences among several surface proteins due to their similar molecular structures and compositions.^[^ ^38^ ^]^ Additionally, with commercially available techniques (e.g., ELISA), it is difficult to achieve the required sensitivity and multiplexing capability in lung‐cancer‐associated sEV profiling. Significantly, to the best of our knowledge, there is no report showing that differences in sEV phenotypes can differentiate between malignant and benign lung nodules.

In summary, we demonstrated the capability of DECODE chip to phenotypically separate sEVs from benign and malignant lung nodules with single sEVs’ precision. To the best of our knowledge, this study is the first to demonstrate a digital single sEV nanotechnology that differentiates benign and malignant lung lesions based on sEV‐specific molecular profiles. DECODE applied an immunoaffinity pulldown of lung‐cancer‐associated sEVs on antibody‐functionalized nanopillar array. Single‐particle‐active SERS nanoboxes that target the expressions of CD63, THBS2, VCAN, and TNC on sEVs were employed to establish specific molecular profiles for identifying malignant and benign phenotypes. We achieved a clinically relevant detection sensitivity of ≈12 EVs µL^−1^ (or 1.25 × 10^2^ sEVs added to DECODE), which can be further improved by scanning more pillars. In an attempt to differentiate benign and malignant lung nodules (*n* = 22) having similar features in clinical imaging, DECODE successfully acquired unique sEV molecular profiles for each patient and classified benign from malignant with an AUC of 0.85. Furthermore, DECODE achieved a complete separation between malignant and healthy participants (*n* = 22) with an AUC of 1.00. We believe that the temporal, biomolecular profiling of sEVs could serve as a triaging tool for screening malignant cases in indeterminate lung nodules. We envisage that the high sensitivity of the DECODE chip could be used to detect minimal residual disease with potential to indicate cancer relapse. This could be particularly important as a relatively high proportion of patients with resected, malignant lung tumors are likely to experience tumor relapse, and thus patient surveillance is indicated.^[^ ^39^ ^]^

## 4 Experimental Section

### Reagents

Hydrogen tetrachloroaurate(III) trihydrate (HAuCl_4_·3H_2_O), silver nitrate (AgNO_3_), MBA, DTNB, TFMBA, MMC, dithiobis (succinimidyl propionate) (DSP), and 11‐mercaptoundecanoic acid (MUA) were obtained from Sigma–Aldrich. Ascorbic acid (AA) was purchased from MP Biomedicals, Inc. 1‐Ethyl‐3‐(3‐dimethylaminopropyl) carbodiimide hydrochloride (EDC) and *N*‐hydroxysulfosuccinimide (Sulfo‐NHS) were bought from Thermo Fisher Scientific. Anti‐CD63 (ab59479) was ordered from Abcam. THSB2 (MAB16351) was purchased from R&D Systems, and VCAN (NBP1‐85432) and TNC (NOVNB11068136) were obtained from Novus Biologicals. THBS2, VCAN, and TNC ELISA kits were bought from Invitrogen (EH452RB), R&D Systems (NBP2‐75354), and Abcam (ab213831), respectively.

### Cell Culture

HCC827, H1975, and HCC78 cell lines were maintained in RPMI‐1640 medium supplemented with 10% fetal calf serum (FCS), 1% GlutaMAX, 100 U mL^−1^ penicillin, and 100 mg mL^−1^ streptomycin, at 37 °C in 5% CO_2_. When cells reached 70–80% confluency, they were washed three times with PBS and seeded into T175 flasks at a split ratio of 1:4 using bovine EV‐depleted media obtained by overnight centrifugation at 100 000 × *g*. Cell culture media were harvested after 72 h.

Normal HBECs (30KT) were cultured in keratinocyte serum‐free medium (KSFM), supplemented with epidermal growth factor (EGF) (5 µg L^−1^) and bovine pituitary extract (50 mg L^−1^), at 37 °C in 5% CO_2_. Bovine pituitary extract was depleted of bovine EVs through overnight centrifugation at 100 000 × *g*. HBECs were grown to 20% confluency, and incubated with EV‐depleted KSFM media for 72 h.

### sEV Purification

Conditioned culture media (CCM) containing EVs were first centrifuged at 800 × *g* for 10 min to remove cells. Following this, CCM was filtered using a 0.22 µm membrane to remove remaining cell debris and large particles. Subsequently, CCM was concentrated to 20 mL and buffer exchanged into PBS with tangential flow filtration using a Minimate capsule with a 300 kDa membrane. The remaining 20 mL was concentrated to 500 µL using a Centricon Plus‐70 Centrifugal Filter with a 100 kDa cutoff, before being purified using size‐exclusion chromatography as previously described.^[^ ^40^ ^]^

Plasma was thawed rapidly and clarified on debris and large vesicles through centrifugation at 1500 × *g* and 10 000 × *g* for 10 and 20 min, respectively. A total of 500 µL of clarified plasma was overlaid on size exclusion columns (Izon) followed by elution with PBS. High EV‐containing fractions were collected as previously described,^[^ ^40^ ^]^ and concentrated to ≈100 µL using Amicon Ultra‐4 10 kDa nominal molecular weight centrifugal filter units.

### sEV Characterization

Purified EVs were characterized for size and tetraspanin (CD9, CD63, and CD81) profiles using nanoFCM. Approximately 5 × 10^8^ particles were labeled with either 20 µL of fluorescein isothiocyanate (FITC) anti‐CD9 (BD Biosciences, clone M‐L13), 0.25 µg of phycoerythrin (PE) anti‐CD63 (Thermo Fisher Scientific, clone H5C6), or 20 µL of allophycocyanin (APC) anti‐CD81 (BD Biosciences, clone JS‐81) for 1 h at room temperature. Stained EVs were subsequently washed once with 1 mL of PBS and purified with the TLA 100.3 rotor using the Optima Max Benchtop Ultracentrifuge at 110 000 × *g* at 4 °C for 30 min. Samples were subsequently resuspended in 50 µL of PBS and analyzed by nanoFCM equipped with 488 and 640 nm lasers. Data were analyzed using FlowJo version 10.8.1.

### Patient Samples/Ethics

Benign and malignant lung samples were obtained through the trial ACTRN12618001789257. All patient blood collection, sEV isolation, and analysis were approved by the QIMR Berghofer Human Ethics committee under p2180. The experiments were carried out with the full, informed consent of the subjects.

### SERS Nanotag Preparation

SERS nanotags were prepared by functionalizing Au–Ag alloy nanoboxes with Raman reporters and antibodies. The nanoboxes were synthesized following the previous publication.^[^ ^23^ ^]^ After that, 1 mL of nanobox solution was centrifuged at 800 × *g* for 15 min and resuspended into 200 µL of H_2_O. Then, 10 µL of 1 mm Raman reporters (i.e., MBA, DTNB, TFMBA, and MMC) and 2 µL of 1 mm MMC were co‐incubated with the nanobox solution at 25 °C for 6 h. After removing the free Raman reporters and MMC, 10 µL of 10 mm EDC and 10 µL of 25 mm sulfo‐NHS were added into the solution at 25 °C, 350 rpm for 30 min to active the carboxyl group. The nanobox solution was centrifuged at 800 × *g* for 15 min to remove the redundant EDC and sulfo‐NHS. Next, 0.5 µg of antibodies (i.e., anti‐CD63, anti‐THBS2, anti‐VACAN, and anti‐TNC) were added into the nanobox solution and incubated at 25 °C, 350 rpm for 30 min. Finally, the SERS nanotags were purified by centrifugation at 800 × *g* for 15 min and dissolved into 0.1% bovine serum albumin (BSA) for storage.

### DECODE Nanofabrication

The nanopillar array of DECODE chip measured 1 mm by 1 mm and contained ≈250 000 pillars. The pillars were 1 µm wide, 1 µm long, and 1 µm deep. Each pillar was separated by 1 µm from adjacent pillars. DECODE was fabricated on a 4 in. silicon wafer using a stepwise process of electron beam lithography, electron beam metal evaporation, and reactive ion etching, as reported previously.^[^ ^23^ ^]^

### DECODE Assay

Prior to use, the DECODE chips were washed with isopropanol, acetone, and water. The gold surface was activated by incubating 10 µL of 5 mm dithiobis (succinimidyl propionate) in dimethyl sulfoxide for 30 min at room temperature. After rinsing of the chip with ethanol, water, and 1× PBS, 10 µL of 10 µg mL^−1^ anti‐TNC antibody (clone 4C8MS, NB110‐68136, Novus Bio) in 1× PBS was incubated overnight at 4 °C. On the next day, the chip was blocked by adding 10 µL of 2% BSA (Sigma–Aldrich) for 1 h at room temperature. Next, the chip was washed three times with 1× PBS. 10 µL of the sEV samples was pipetted on the chip, incubated for 30 min at room temperature, and washed three times with washing buffer (0.01% Tween‐80, 0.1% BSA, 1× PBS). Subsequently, 10 µL of SERS nanoboxes was incubated on the chip for 30 min at room temperature, washed three times with washing buffer, and stored at 4 °C prior to Raman mapping.

Raman mapping was performed using a Witec Alpha 300 R microscope with an EMCCD camera, 100× objective (numercial aperture, NA = 0.9), and the parameters as follows: He–Ne laser, 632.8 nm; laser power, 35 mW; grating, 600 g mm^−1^; spectral resolution, 1.390–2.114 cm^−1^; integration time, 0.01 s; scanning area size, 60 µm  ×  48 µm; scanning area resolution, 86 points per line, and 69 lines per image. Initial calibration of the system was performed by measuring the intensity of the Si peak at 520 cm^−1^ using a Si wafer. To map the pillar array, the objective was first focused on the Si substrate. Subsequently, the 100× objective was lifted by 1 µm along the *z*‐axis and the SERS mapping was started. Nine areas were scanned to 60 µm  ×  48 µm per pillar array. This corresponded to scanning a total of 6580 pillars.

## Conflict of Interest

The authors declare no conflict of interest.

## Supporting information

Supporting Information

## Data Availability

The data that support the findings of this study are available from the corresponding author upon reasonable request.
