# Supplementary material for: Digital Decoding of Single Extracellular Vesicle Phenotype Differentiates Early Malignant and Benign Lung Lesions
Source: Adv Sci (Weinh). 2022 Nov 17;10(1):2204207. doi: 10.1002/advs.202204207 (PMC9811438; doi:10.1002/advs.202204207)
Supplement: Supplementary file 1 — Supporting Information [file ADVS-10-2204207-s001.pdf]

ADVANCED SCIENCE
Open Access

Supporting Information

for *Adv. Sci.*, DOI 10.1002/advs.202204207

Digital Decoding of Single Extracellular Vesicle Phenotype Differentiates Early Malignant and Benign Lung Lesions

Junrong Li, Abu A. I. Sina, Fiach Antaw, David Fielding, Andreas Möller, Richard Lobb, Alain Wuethrich* and Matt Trau**

Supplementary Information

Digital decoding of single extracellular vesicle phenotype differentiates early malignant and benign lung lesions

Junrong Li[1], Abu A. I. Sina[1], Fiach Antaw[1], David Fielding[2], Andreas Moeller[3], Richard Lobb[1]*, Alain Wuethrich[1]*, and Matt Trau[1,4]*

[1]Centre for Personalised Nanomedicine, Australian Institute for Bioengineering and Nanotechnology (AIBN), The University of Queensland, Brisbane, QLD 4072, Australia.

[2]Dept of Thoracic Medicine, Royal Brisbane and Women's Hospital, Herston, QLD, Australia

[3]Tumour Microenvironment Laboratory, QIMR Berghofer Medical Research Institute, Herston, Queensland 4006, Australia

[4]School of Chemistry and Molecular Biosciences, The University of Queensland, Brisbane, QLD 4072, Australia.

*Corresponding authors

*Email: richard.lobb@uq.edu.au; a.wuethrich@uq.edu.au; m.trau@uq.edu.au.

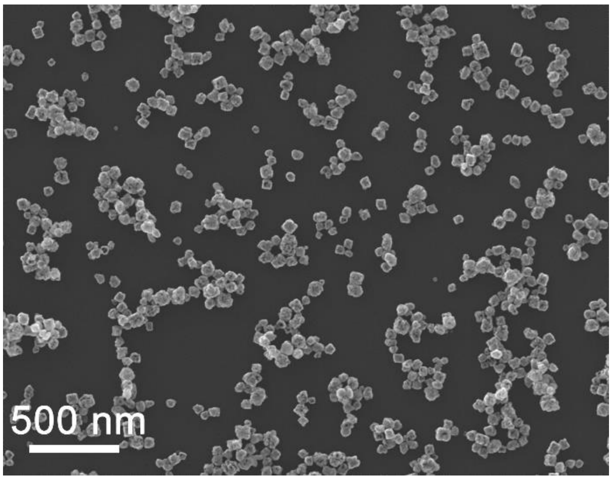

Figure S1. SEM image of the prepared gold-silver alloy nanoboxes.

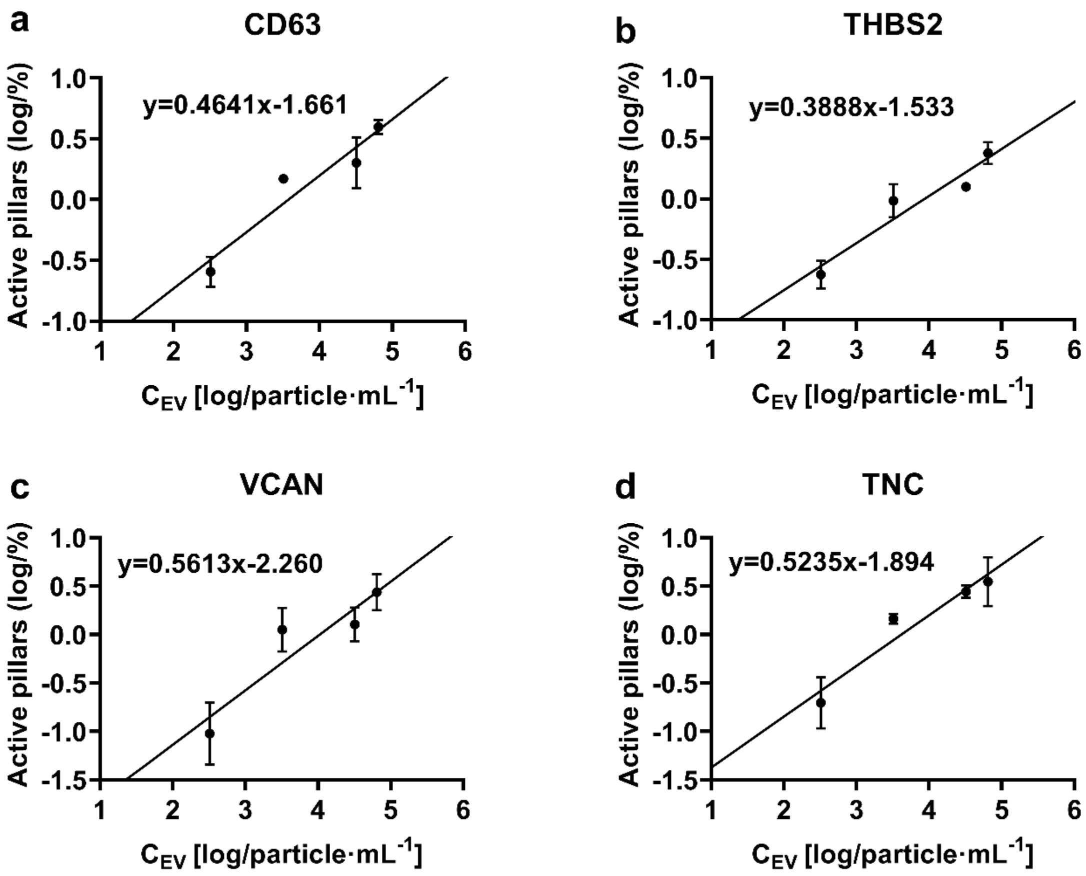

Figure S2. Sensitivity for the simultaneous profiling of four biomarker on sEV surfaces. Linear relationship curve for the detection of (a) CD 63, (b) THBS2, (c) VCAN, and (d) TNC. The error bars represented the standard deviation from three independent technical measurements on three nanopillar arrays.

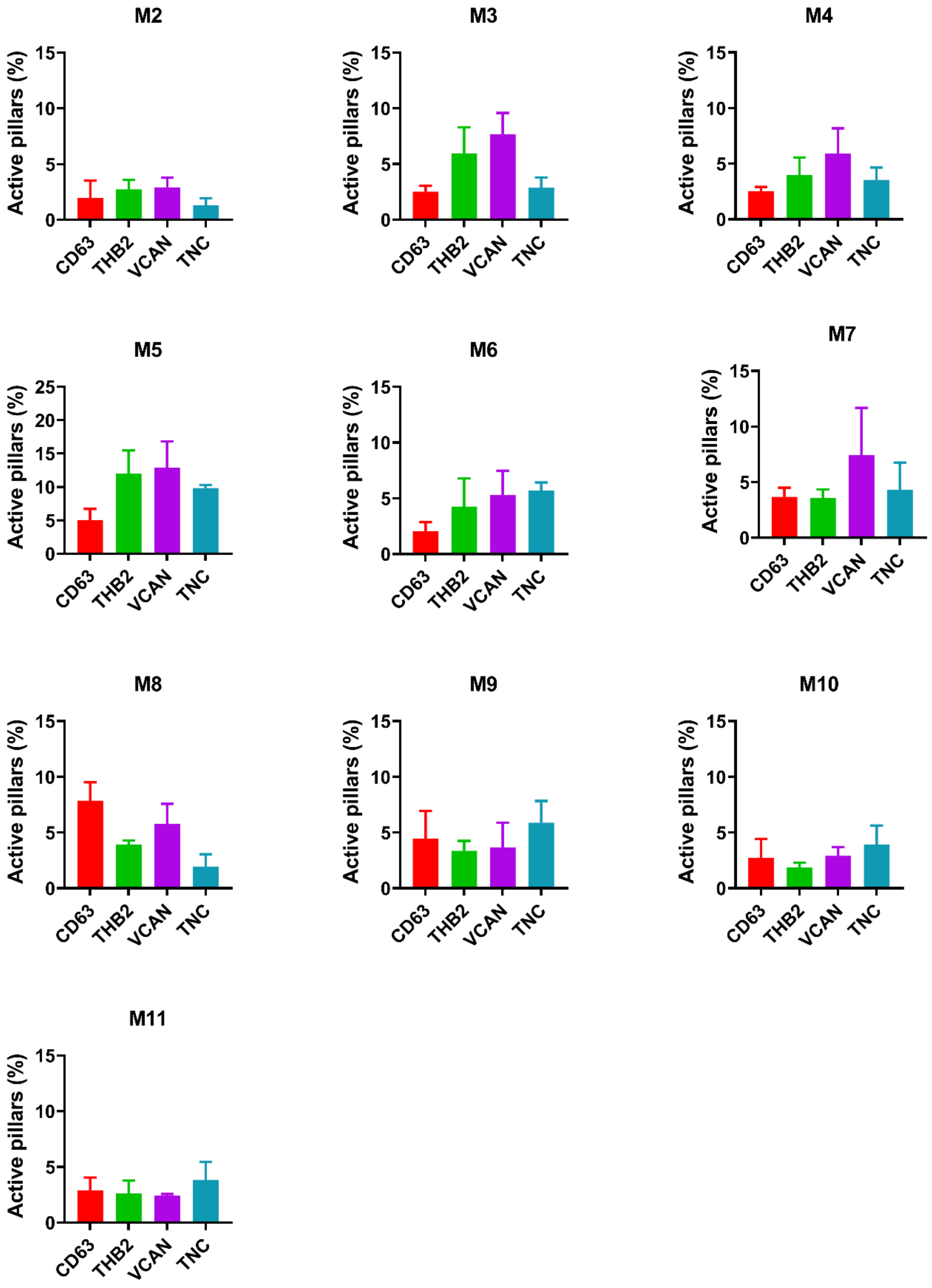

Figure S3. DECODE phenotyping clinical plasma derived sEVs. Molecular profiles of sEVs derived from patients with malignant lung nodules (M2-M11).

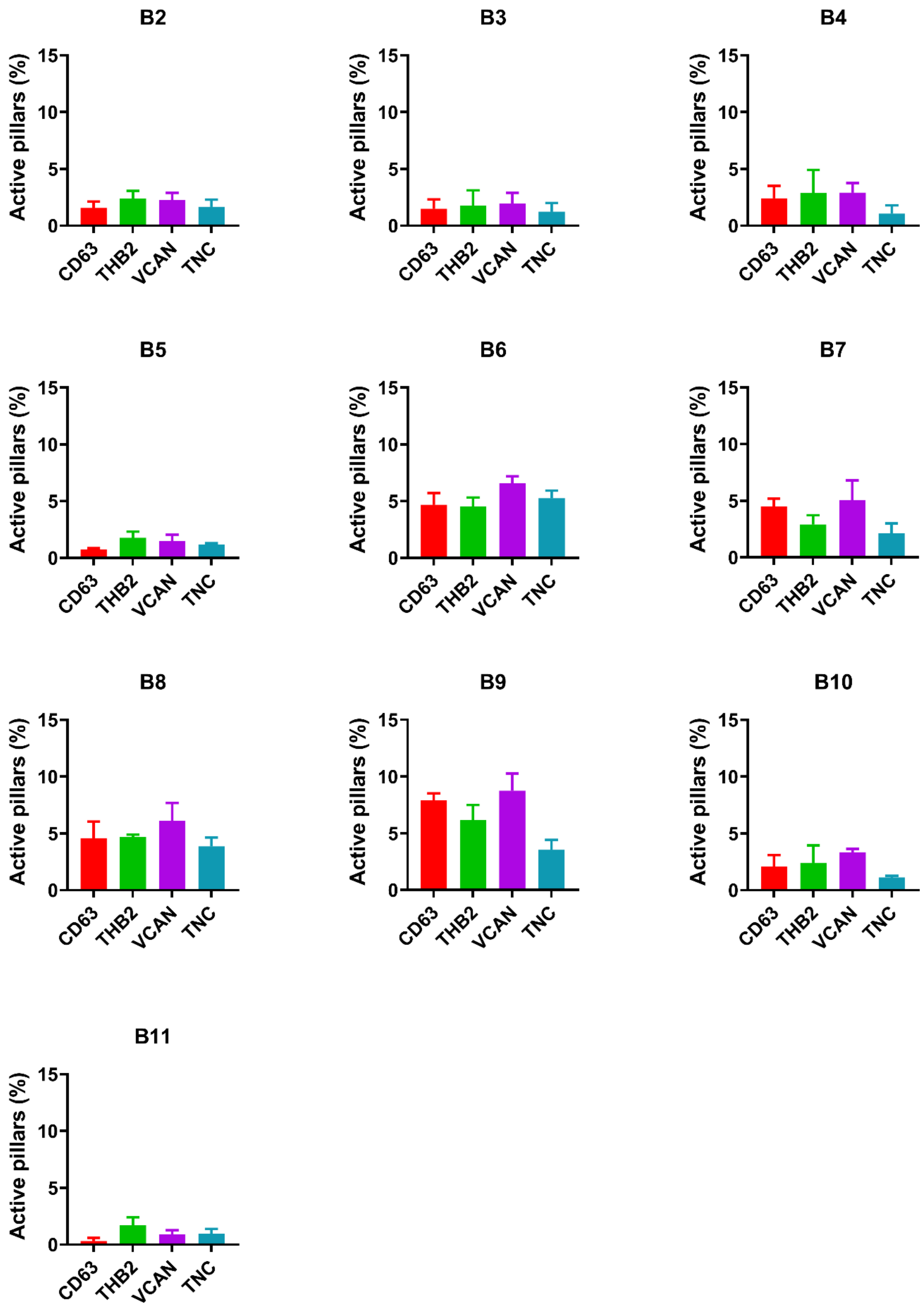

Figure S4. DECODE phenotyping clinical plasma derived sEVs. Molecular profiles of sEVs derived from patients with benign lung nodules (B2-B11).

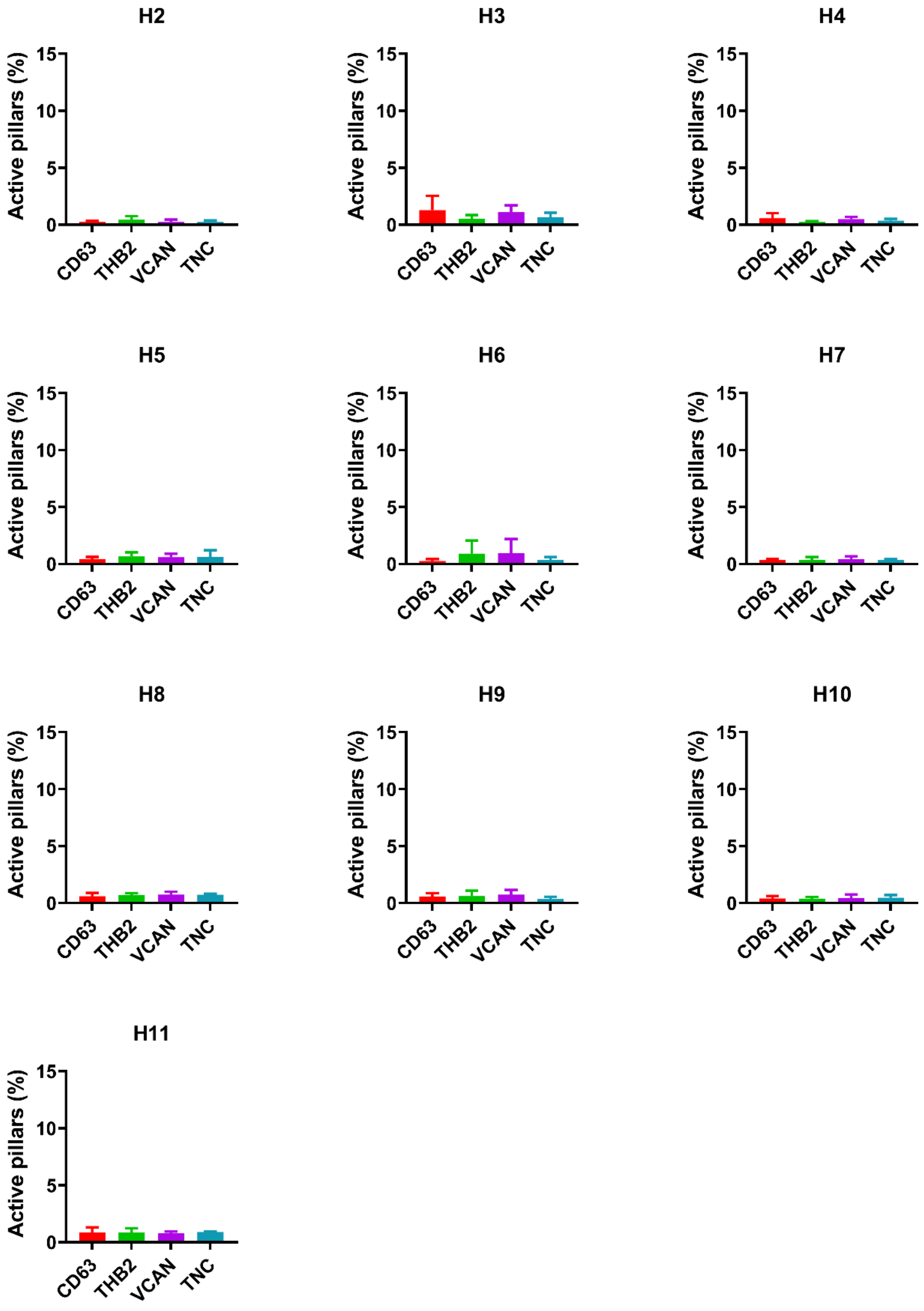

Figure S5. DECODE phenotyping clinical plasma derived sEVs. Molecular profiles of sEVs derived from healthy participants (H2-H11).
